# Cell Death in Acute Organ Injury and Fibrosis

**DOI:** 10.3390/ijms25073930

**Published:** 2024-04-01

**Authors:** Taro Yasuma, Esteban C. Gabazza

**Affiliations:** 1 Department of Immunology, Mie University Faculty and Graduate School of Medicine, Edobashi 2-174, Tsu 514-8507, Japan; t-yasuma0630@clin.medic.mie-u.ac.jp; 2 Department of Diabetes and Endocrinology, Mie University Faculty and Graduate School of Medicine, Edobashi 2-174, Tsu 514-8507, Japan

Tissue fibrosis is characterized by the excessive accumulation of extracellular matrix in various organs, including the lungs, liver, skin, kidneys, pancreas, and heart, ultimately leading to organ failure [1,2]. This fibrotic process may be triggered by tissue injury resulting from diverse mechanisms such as infection, trauma, metabolic disorders, wounds, acute or chronic inflammation, autoimmune disorders, cancer, or unknown mechanisms [1]. Following injury, an abnormal tissue repair mechanism ensues, marked by the enhanced accumulation of fibroblasts and/or myofibroblasts within the affected organ, which overproduce extracellular matrix proteins [3]. Various cell types, including parenchymal epithelial cells, vascular endothelial cells, and cells from the innate or acquired immune systems, participate in this fibrotic process by secreting factors that recruit and activate fibroblasts to produce extracellular matrix proteins [4].

Importantly, during tissue injury and fibrosis, parenchymal cells undergo cell death, leading to their replacement by collagen-producing cells, thereby exacerbating the fibrotic process [5,6,7,8]. Profibrotic cytokines such as transforming growth factor-β1 (TGF-β1) promote the production and secretion of extracellular matrix proteins and may also cause parenchymal cell apoptosis, further contributing to tissue sclerosis [9]. TGF-β1 has been reported to induce alveolar epithelial cell apoptosis in lung fibrosis and hepatocytes in liver cirrhosis [10]. Increased levels of TGF-β1 have also been implicated in the pathogenesis of fibrosis in several organs in patients with diabetes mellitus [11,12]. TGF-β1 may induce the apoptosis of insulin-producing β-cells, increase insulin resistance contributing to the acceleration of diabetes mellitus, or induce the apoptosis of podocytes and renal tubular epithelial cells contributing to the pathogenesis of diabetic nephropathy [9,11].

Cell death is indispensable for numerous physiological processes, including embryonic development, tissue homeostasis, and immune responses [13]. During embryogenesis, apoptosis eliminates superfluous cells, shapes developing tissues, and regulates organ morphogenesis [13,14]. In adult organisms, programmed cell death maintains tissue integrity by eliminating damaged or senescent cells, thereby preventing the accumulation of potentially harmful cellular debris. Moreover, cell death serves as a defense mechanism against pathogens, facilitating the clearance of infected cells and promoting immune surveillance [14]. Cell death can occur through several mechanisms including apoptosis, pyroptosis, necrosis, and autophagy [13].

The death of parenchymal cells has been implicated in the pathogenesis of organ fibrosis [5,15]. Dead cells are replaced by fibroblasts, which perpetuate fibrosis by producing and releasing extracellular matrix proteins [5]. Furthermore, the progression of fibrosis is often preceded by acute tissue injury crises, such as acute exacerbation of interstitial lung disease and acute kidney injury, which are associated with an increased death of parenchymal and vascular endothelial cells [16,17,18].

The amelioration of organ injury/fibrosis by inhibitors of apoptosis or pyroptosis further supports the implication of cell death in the pathogenesis of tissue injury/fibrosis [19,20]. For example, the inhibition of pyroptosis of bladder epithelial cells improves neurogenic bladder fibrosis, lung overexpression of matrix metalloproteinase-2 has been shown to mitigate lung fibrosis by blocking apoptosis of lung epithelial cells, and selective cannabinoid type II receptors, which are known to block apoptosis, protect against inflammatory response in endotoxin-induced acute lung injury and hepatic ischemic/reperfusion injury [21,22,23,24,25].

The resistance of fibroblasts or myofibroblasts to cell death has also been reported to contribute to the progression of fibrosis [26]. Some drugs undergoing clinical trials accelerate the apoptosis of myofibroblasts [27,28,29]. However, it is worth noting that the injury or apoptosis of fibroblasts/myofibroblasts may also be detrimental under certain environmental or pathological conditions. For example, excessive mechanical stress, aging, or hypoxia can cause sublethal injury or the apoptosis of anterior cruciate ligament fibroblasts, hindering fibroblast cell motility and ligament regeneration [30]. An improvement in fibroblast motility or survival has been shown to protect and accelerate the healing of the anterior cruciate ligament [31,32,33]. In addition, components of extracellular matrix proteins may also be important to protect some organ normal resident cells from injury and apoptosis. For example, a previous study has shown that the presence of elastin in the skin may be important to protect against the loss of melanocytes in vitiligo [34]. Apoptosis caused by immune cells is involved in the loss of melanocytes in vitiligo conditions [35].

In summary, tissue fibrosis, triggered by various insults, involves excessive extracellular matrix accumulation leading to organ dysfunction. Profibrotic cytokines such as TGF-β1 promote matrix production and the apoptosis of parenchymal cells, worsening fibrosis. Cell death, vital for development and immunity, contributes to fibrosis progression by replacing dead cells with collagen-producing fibroblasts. Strategies targeting cell death pathways offer potential in mitigating fibrosis, but caution is needed due to its dual role in tissue repair and pathology.
